# Association of the systemic immune-inflammation index (SII) and severity of diabetic ketoacidosis in patients with type 1 diabetes mellitus: a retrospective cohort study

**DOI:** 10.1097/MS9.0000000000002185

**Published:** 2024-05-20

**Authors:** Mohamed Aon, Ahmed H. Aoun, Ahmad Alshami, Abdulrahman Alharbi, Fahed Alshammari, Mohamad Alnajjar, Ahmad Almutawtah, Bader Bin Naji, Abdullateef Alsaeed, Omar A. Abdelwahab

**Affiliations:** Departments ofa Internal Medicine; b Pediatrics, Faculty of Medicine, Cairo University, Giza; c Faculty of Medicine, Al-Azhar University, Cairo, Egypt; d Primary Health Care Corporation, Doha, Qatar; e Department of Internal Medicine, Jahra Hospital, Jahra, Kuwait

**Keywords:** complete blood count, diabetes mellitus, diabetic ketoacidosis, neutrophil-lymphocyte ratio, platelet-lymphocyte ratio, systemic immune-inflammation index

## Abstract

**Background:**

Diabetic ketoacidosis (DKA) is the most serious metabolic complication of type 1 diabetes mellitus (T1DM). Insulin deficiency and inflammation play a role in the pathogenesis of DKA. The authors aimed to assess the systemic immune-inflammation index (SII) as a marker of severity among T1DM patients with DKA and without infection.

**Methods:**

The authors included T1DM patients older than or equal to 12 years hospitalized because of DKA. The authors excluded patients with infection or any condition that can change SII parameters or cause metabolic acidosis. The authors compared SII, neutrophil-lymphocyte ratio (NLR), and platelet-lymphocyte ratio (PLR) between severe and non-severe DKA groups. The authors also assessed the need for an ICU, length of stay, and 90-day readmission rate between the groups.

**Results:**

The study included 241 patients with a median age of 17 (14, 24) years, and 44.8% were males. More patients with severe DKA (45%) required ICU admission (*P*<0.001). Median SII increased with DKA severity, and the difference was significant (*P*=0.033). No significant difference was observed as regards median NLR or PLR (*P*=0.380 and 0.852, respectively). SII, but not NLR or PLR, had a significant negative correlation with PH (r=−0.197, *P*=0.002) and HCO_3_ level (r=−0.144, *P*=0.026). Also, being in the highest SII quartile was an independent risk factor for DKA severity (OR, 2.522; 95% CI, 1.063–6.08; *P*=0.037). The authors estimated an SII cut-off value of 2524.24 to predict DKA severity with high specificity.

**Conclusion:**

Elevated SII is a risk factor for DKA severity in T1DM. It is better than NLR and PLR in prognosticating DKA patients. These findings highlight the role of inflammation in DKA. SII can help as a valuable and simple tool to assess DKA severity.

## Introduction

Highlights

- Systemic immune-inflammation index (SII) is a convenient and easily measured index of diabetic ketoacidosis (DKA) severity.
- SII correlates with the degree of metabolic acidosis.
- A higher SII quartile is an independent risk factor of DKA severity.

Diabetic ketoacidosis (DKA) is one of the most severe acute metabolic complications of diabetes mellitus (DM). It occurs mainly among patients with type 1 DM (T1DM), but under the circumstances of extreme stress, DKA may also occur in type 2 DM (T2DM)^1^. The presence of hyperglycemia (>11 mmol/l), ketonemia (>3 mmol/l) or ketonuria (2+ or more), and acidosis [PH<7.3 or bicarbonate (HCO_3_)<15–18 mmol/l] are the essential criteria to diagnose DKA^2,3^. DKA is responsible for a huge burden of morbidity and medical expenses among patients with DM. Mortality is usually less than 1%, but may exceed 13% if diagnosis and management is delayed. Therefore, DKA patients require prompt treatment, and any delay in identifying severe DKA cases can lead to worse outcomes^4^. The key criteria to define severe DKA are PH less than 7.1 or HCO_3_ less than 5 mmol/l. Additionally, disturbed level of consciousness, unstable vital signs, and marked elevation of anion gap (AG) are indicators of severity. This reflects the severe metabolic derangements that occur with DKA^2,3^. In addition to the acute metabolic disturbances, DKA provokes a systemic inflammatory response through increased levels of various cytokines such as interleukin (IL)-8, IL-6, IL-10, tumor necrosis factor-alpha (TNF-α), and IL-1B. This will lead to cellular activation, cellular adhesion, increased oxidative stress, and endothelial damage, possibly contributing to complications^5,6^. Consequently, surrogate markers of inflammation and immune status may help in the early identification of patients with severe DKA.

The complete blood count (CBC), being a proxy marker of inflammation and immune status, has gained interest as a potential marker of DKA severity being an inexpensive, easily available, and simple test. Earlier studies have demonstrated higher counts of total white blood cells (WBCs) and neutrophils among patients with DKA compared to non-DKA diabetic patients and non-diabetic controls. However, the results were limited by the presence of infections and the non-specificity of leukocytosis as a marker of acute illness^7^. Combined markers that can be obtained from CBC, such as neutrophil-lymphocyte ratio (NLR) and platelet-lymphocyte ratio (PLR), may have a better prognostic value compared to single cell counts. A recent study found a higher NLR among uninfected adult T1DM patients with DKA^8^. Similar findings were demonstrated among pediatric T1DM patients where NLR increased progressively with DKA severity^9^. Increased PLR was linked to diabetes complications such as diabetic nephropathy and retinopathy^10,11^.

The systemic immune-inflammation index (SII), originally described by Hu and his colleagues, had a better prognostic value compared to NLR and PLR among cancer patients^12,13^. Recently, studies have suggested a link between SII and increased risk of atherosclerotic cardiovascular disease (ASCVD)^14^, hepatic steatosis^15^, and worse outcomes among hypertensive patients and patients with stroke^16,17^.

To the best of our knowledge, SII was not assessed before as a marker of severity in DKA. Therefore, this study aims to examine SII as a marker of severity in T1DM patients with DKA in an uninfected state.

## Methods

### Study subjects and design

This study included patients hospitalized between 1 August 2021 and 31 December 2022 in the main district hospital. Patients’ demographic, clinical, and laboratory data on admission were retrieved from the electronic medical records system. Laboratory parameters recorded on admission included serum glucose, PH, HCO_3_, AG, potassium, urea, creatinine, alanine transaminase (ALT), aspartate transaminase (AST), glycated hemoglobin (HbA1c), procalcitonin (PCT), and CBC. The eligibility criteria were: (1) diagnosis of T1DM; (2) hospitalization because of DKA; (3) age older than or equal to 12 years. The exclusion criteria were: (1) diagnosis of T2DM; (2) infection; (3) renal impairment; (4) malignancy; (5) ASCVD; (6) history of any medical condition or medications that can change CBC parameters or cause metabolic acidosis; (7) pregnancy. Assuming an outcome incidence of 31% in the unexposed group with an assumed relative risk of 1.88^18^, at least 49 patients will be needed in each group to achieve a study power of 80% with 95% confidence level.

### Exposure and outcome variables

Patients were divided into groups according to DKA severity. The criteria to diagnose DKA and determine DKA severity were mentioned previously in the introduction section^2,3^. We compared SII, NLR, and PLR between the groups. The neutrophil, platelet, and lymphocyte counts were performed using an automated blood cell analyzer (Sysmex) and expressed as (*10^9^/l cells). SII, NLR, and PLR were calculated using the following formulas:

Also, we compared the need for ICU admission, 90-day readmission rates, and length of hospital stay between the groups.

### Statistical analysis

We expressed categorical variables as frequencies and percentages (%), while continuous variables were expressed as the median and interquartile range (IQR) since most of the data were not normally distributed. Difference between the groups was assessed using the χ^2^ test for categorical variables, and the Kruskal–Wallis test or one-way ANOVA test for continuous variables, as appropriate. We used multivariate logistic regression analysis to calculate the odds ratio (OR) and 95% CI for the association between SII and DKA severity. We included variables in the multivariate analysis models if they had statistical significance in the univariate analysis or were clinically relevant to DKA severity. We designed three models to control the confounding variables. Model 1, was unadjusted; model 2, was adjusted to age, sex, and comorbidities; and model 3, was adjusted to age, sex, comorbidities, blood glucose, HbA1c, creatinine, urea, AST, ALT, and PCT. We used the Spearman correlation to find the relationship between the peripheral blood ratios and the metabolic parameters. We drafted the receiver operating characteristic (ROC) curve to find the best cut-off value for SII, NLR, and PLR that can predict severe DKA. We analyzed data using SPSS version 26.0 (SPSS Inc.), and we considered *P* less than 0.05 to be statistically significant.

This research was reported in line with Strengthening the Reporting of Cohort Studies in Surgery Statement (STROCCS, Supplemental Digital Content 1, http://links.lww.com/MS9/A481)^19^.

In accordance with the Declaration of Helsinki, the study is registered at https://www.clinicaltrials.gov/ with registration ID (NCT06251895)^20^.

The study was approved by Jahra Hospital research committee with approval number (J1–18072023).

## Results

### Baseline characteristics of participants

**Figure 1:**
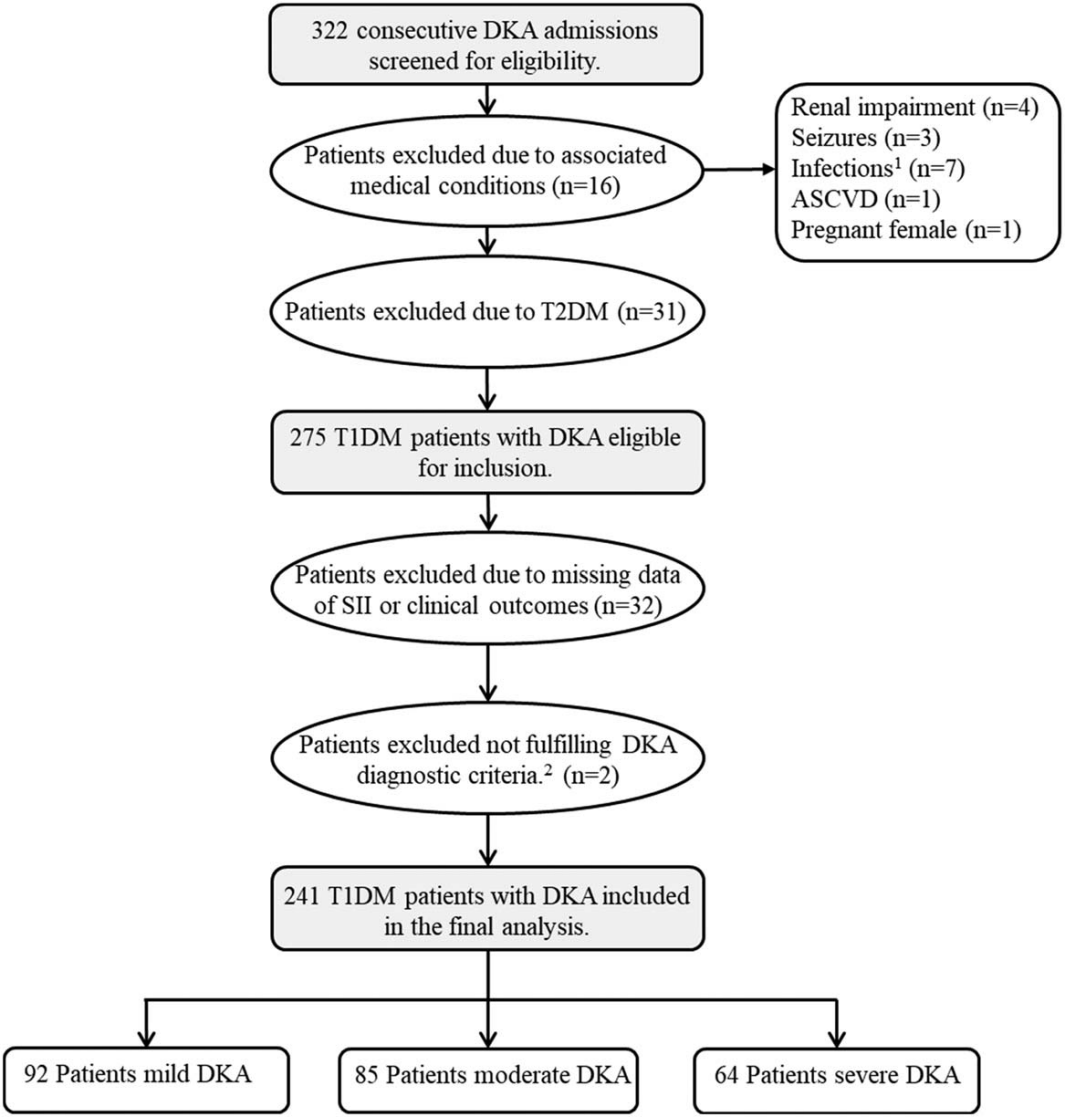
The flowchart for the selection of the study population. 1 Diagnosis based on positive cultures from clinically significant sites (*n*=4) and/or fever with clinically compatible symptoms (*n*=3). 2 Patients who were labeled in the electronic medical records system as DKA, but after reviewing their laboratory parameters they did not fulfill the diagnostic criteria of DKA. ASCVD, atherosclerotic cardiovascular disease; DKA, diabetic ketoacidosis; SII, systemic immune-inflammation index; T1DM, type 1 diabetes mellitus; T2DM, type 2 diabetes mellitus.

Initially, we screened 322 consecutive DKA admissions for eligibility. After the application of inclusion and exclusion criteria, 241 patients were included in the final analysis. (Fig. 1).

The study included 133 female patients (55.2%) and 108 male patients (44.8%). The median age of participants was 17 (14, 24) years. The most observed comorbidity was hypothyroidism in 2.9% of patients. Table 1 demonstrates baseline clinical and laboratory data of the study population. There was no substantial difference in age, sex, comorbidities, or background glycemic control between the groups (*P*=0.212, 0.403, 0.273, and 0.130, respectively). Neutrophilic leukocytosis was more prevalent among patients with severe DKA (*P*<0.001). The median platelet and urea levels were higher among patients with severe DKA, although still within the normal range (*P*=0.001 and 0.020, respectively).

**Table 1:** Baseline characteristics and clinical outcomes of the study population according to DKA severity

	Mild DKA (*n*= 92)	Moderate DKA (*n*= 85)	Severe DKA (*n*= 64)	*P*
Baseline demographic and clinical data				
Age (year)	18 (15, 24)	17 (15, 26)	17 (14, 20)	0.212
Sex, *n* (%)				0.403
Female	53 (57.6)	42 (49.4)	38 (59.4)	
Male	39 (42.4)	43 (50.6)	26 (40.6)	
Comorbidities, *n* (%)				0.273
Hypothyroidism	5 (5.4)	0	2 (3.1)	
Others^a^	3 (3.3)	2 (2.4)	1 (1.6)	
SBP (mmHg)	115 (108, 123)	113 (106, 120)	116 (106, 122)	0.726
DBP (mmHg)	70 (67, 76)	70 (64, 74)	68 (61, 72)	**0.021**
Pulse (bpm)	84 (78, 88)	89 (78, 100)	91 (80, 106)	**0.015**
Temperature (^o^C)	37.0 (36.8, 37.0)	37.0 (36.7, 37.0)	36.9 (36.6, 37.0)	0.420
Baseline laboratory data				
Glucose (mmol/l)	23 (17, 29)	27 (22, 34)	27 (20, 33)	**0.006**
HbA1c (%)	11.40 (10.10, 12.70)	11.60 (10.65, 13.10)	12.40 (10.70, 13.10)	0.130
PH	7.26 (7.23, 7.28)	7.16 (7.12, 7.18)	7.04 (6.98, 7.07)	**<0.001**
AG	21 (17, 25)	26 (23, 32)	30 (26, 34)	**<0.001**
HCO_3_ (mmol/l)	15.8 (14.2, 17.3)	11.7 (10.6, 13.0)	9.1 (7.6, 9.7)	**<0.001**
Ketones (mmol/l)	3.40 (3.10, 4.65)	4.50 (3.95, 5.60)	4.30 (3.90, 5.10)	**<0.001**
Potassium (mmol/l)	4.40 (4.00, 4.70)	4.70 (4.20, 5.10)	4.75 (4.18, 5.40)	**<0.001**
WBCs (*10^9^/l)	8.9 (7.4, 11.8)	10.0 (8.2, 12.0)	12.1 (9.7, 16.9)	**<0.001**
Neutrophils (*10^9^/l)	6.2 (4.9, 9.0)	7.0 (5.2, 9.7)	9.4 (6.4, 12.9)	**<0.001**
Lymphocytes (*10^9^/l)	2.10 (1.40, 2.80)	2.30 (1.60, 3.17)	2.41 (1.55, 3.32)	0.195
Hemoglobin (g/l)	140 (120, 152)	146 (131, 158)	146 (123, 158)	0.054
Platelets (*10^9^/l)	340 (276, 413)	368 (303, 429)	417 (325, 471)	**0.001**
Urea (mmol/l)	4.45 (3.27, 5.32)	5.00 (3.90, 6.40)	5.10 (3.95, 6.03)	**0.020**
Creatinine (umol/l)	47 (41, 57)	52 (42, 65)	50 (40, 67)	0.250
AST (IU/l)	17 (15, 24)	18 (15, 26)	22 (15, 28)	0.073
ALT (IU/l)	16 (12, 23)	18 (14, 25)	19 (13, 29)	0.240
PCT (ng/ml)	0.06 (0.05, 0.32)	0.12 (0.05, 0.27)	0.14 (0.06, 0.34)	0.170
Clinical outcomes				
ICU admission				**<0.001**
Yes, *n* (%)	4 (4.3)	8 (9.4)	29 (45.3)	
No, *n* (%)	88 (95.7)	77 (90.6)	35 (54.7)	
LoS (day)	2 (1, 3.25)	2 (2, 3)	2 (2, 4)	0.568
90-days readmission, *n* (%)				0.211
Yes	11 (12)	18 (21.2)	13 (20.3)	
No	81 (88)	67 (78.8)	51 (79.7)	

Bold values are statically significant of *P* value < 0.05.

Variables are expressed as median (IQR) or number (%).

AG, anion gap; ALT, alanine transaminase; AST, aspartate transaminase; DBP, diastolic blood pressure; DKA, diabetic ketoacidosis; HbA1c, glycated hemoglobin; HCO_3_, bicarbonate; IQR, interquartile range; LoS, length of hospital stay; PCT, procalcitonin; SBP, systolic blood pressure; WBCs, white blood cells.

^a^ Other comorbidities include celiac disease (*n*=2), Grave’s disease (*n*=1), hypertension (*n*=1), hyperlipidemia (*n*=1), and growth hormone deficiency (*n*=1).

More patients in the severe DKA group required ICU admission (45%) compared to mild (4.3%) and moderate (9.4%) DKA cases, and the difference was significant (*P*<0.001). On the contrary, no significant difference was noticed as regards length of hospital stay or 90-day readmission rate (*P*=0.568 and 0.211, respectively). (Table 1) In-hospital mortality did not occur among any of the participants in all groups.

### Association between SII and DKA severity

**Figure 2:**
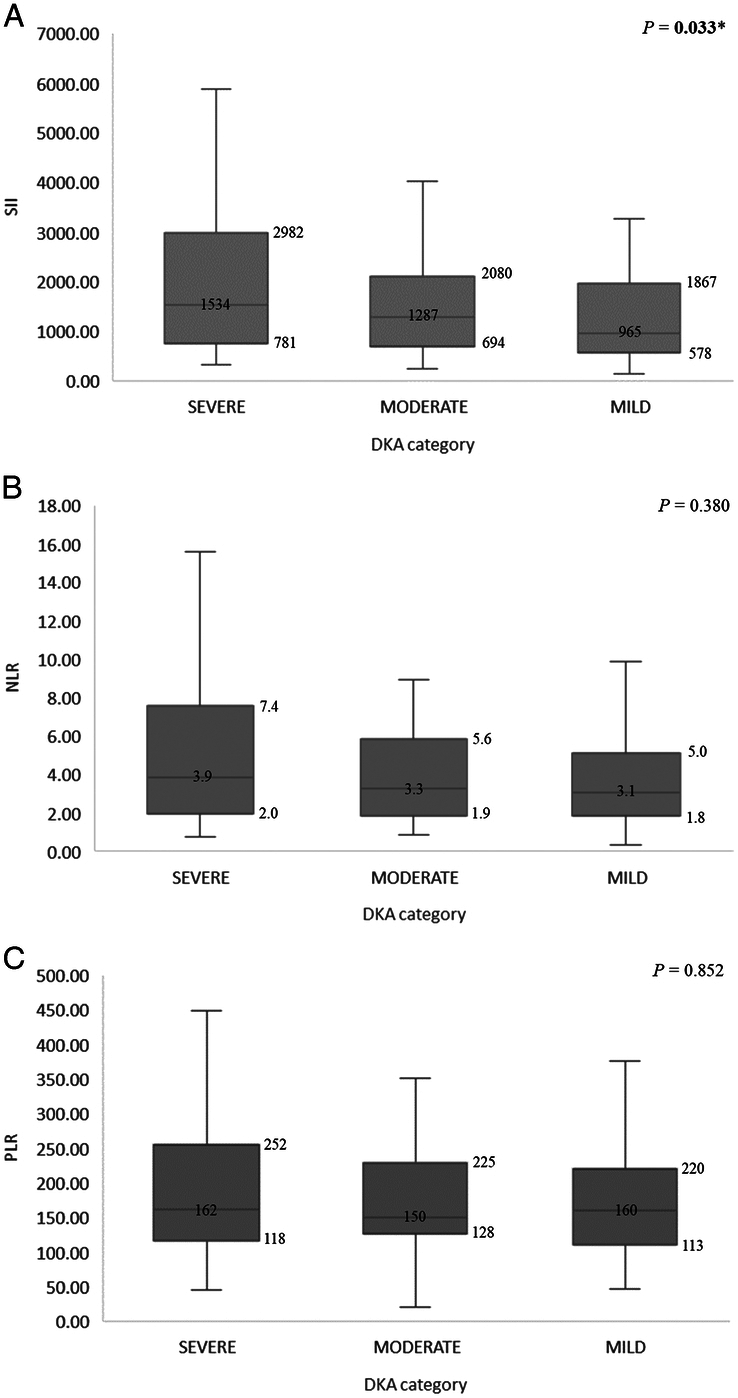
The difference in peripheral blood ratios according to DKA severity groups: SII (A), NLR (B), and PLR (C). The numbers on the box and whisker diagram represent the median and IQR. * *P*<0.05. DKA, diabetic ketoacidosis; IQR, interquartile range; NLR, neutrophil-lymphocyte ratio; PLR, platelet-lymphocyte ratio; SII, systemic immune-inflammation index.

As regards the assessed peripheral blood ratios, SII increased progressively with increasing DKA severity, and the difference between groups was statistically significant (*P*=0.033). No significant difference between severe and non-severe DKA was seen as regards NLR or PLR (*P*=0.380 and 0.852, respectively). (Fig. 2).

### Correlation analysis between SII and DKA severity parameters

**Figure 3:**
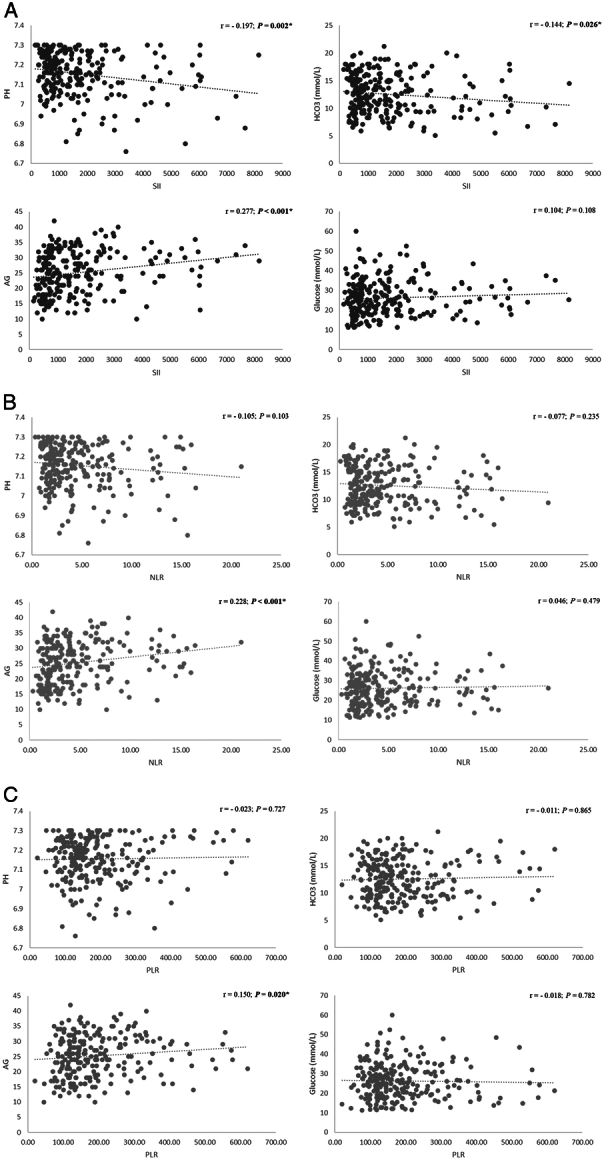
Correlation scatter plot for the relation between the metabolic parameters and the peripheral blood ratios: SII (A), NLR (B), and PLR (C). * *P*<0.05. AG, anion gap; HCO3, bicarbonate; NLR, neutrophil-lymphocyte ratio; PLR, platelet-lymphocyte ratio; SII, systemic immune-inflammation index.

Figure 3 shows the correlation between the different peripheral blood ratios and the metabolic parameters in our study. We found that all tested blood ratios (SII, NLR, and PLR) had a significant positive correlation with AG (*P* < 0.001, < 0.001, and 0.020, respectively). However, only SII had a significant negative correlation with PH (r=- 0.197, *P*=0.002) and HCO_3_ level (r=-0.144, *P*=0.026). None of the ratios had a significant correlation with glucose levels.

### SII as a risk factor for DKA severity

We converted SII into a categorical variable (quartiles) and used regression analysis to assess the independent effect of SII on DKA severity. Table 2 demonstrates that being in the highest SII quartile was an independent risk factor for DKA severity. This association was significant in the unadjusted model (OR, 2.18; 95% CI, 1.120–4.28, *P*=0.023) and remained significant in the adjusted models (OR, 2.123; 95% CI, 1.083–4.20, *P*=0.029; OR, 2.522; 95% CI: 1.063–6.08, *P*=0.037, for model 2 and 3, respectively). Higher NLR and PLR quartiles were not risk factors for DKA severity as shown in supplementary table 1, Supplemental Digital Content 2, http://links.lww.com/MS9/A482.

**Table 2:** Association between SII and DKA severity by regression analysis

SII quartile	OR (95% CI)	*P*
Model 1		
Q1	Reference (reference)	Reference
Q2	1.00 (0.513–1.95)	1.00
Q3	1.60 (0.826–3.11)	0.165
Q4	2.18 (1.120–4.28)	**0.023**
Model 2		
Q1	Reference (reference)	Reference
Q2	—	—
Q3	—	—
Q4	2.123 (1.083–4.20)	**0.029**
Model 3		
Q1	Reference (reference)	Reference
Q2	—	—
Q3	—	—
Q4	2.522 (1.063–6.08)	**0.037**

Bold values are statically significant of *P* value < 0.05.

SII quartiles: Q1<624 (*n*= 61); Q2=624–1226 (*n*= 60); Q3=1227–2104 (*n*= 60); Q4 ≥ 2105 (*n*= 60).

Model 1: Unadjusted odds ratio (OR).

Model 2: Adjusted to age, sex, and comorbidities.

Model 3: Adjusted to age, sex, comorbidities, blood glucose, HbA1c, creatinine, urea, AST, ALT, and PCT.

ALT, alanine transaminase; AST, aspartate transaminase; DKA, Diabetic ketoacidosis; HbA1c, glycated hemoglobin; OR, odds ratio; PCT, procalcitonin; SII, systemic immune-inflammation index.

### ROC curve analysis

The diagnostic accuracy of SII, NLR, and PLR in predicting severe DKA was investigated by the ROC curve. As shown in Table 3, the best predictor of DKA severity among them was SII, and the best SII statistical cut-off value to predict severe DKA was 2524.24 with a specificity of 85.3% and a sensitivity of 34.4%. (supplementary figure 1, Supplemental Digital Content 3, http://links.lww.com/MS9/A483).

**Table 3:** ROC curve AUC and cut-off values to predict DKA severity.

Variable	AUC	95% CI	*P*	Cut-off value	Sensitivity (%)	Specificity (%)
SII	0.594	0.510–0.678	**0.026**	2524.24	34.4	85.3
NLR	0.555	0.471–0.640	0.189	5.65	37.5	78.0
PLR	0.521	0.437–0.605	0.618	242.83	29.7	79.1

Bold values are statically significant of *P* value < 0.05.

AUC, area under the curve; DKA, Diabetic ketoacidosis; NLR, neutrophil-lymphocyte ratio; PLR, platelet-lymphocyte ratio; ROC, receiver operating characteristics; SII, systemic immune-inflammation index.

## Discussion

In the current study, we found that higher SII was more prevalent in T1DM patients with severe DKA without infection. We also identified a significant negative correlation between SII and both PH and HCO_3_ levels, which are the main parameters used to decide DKA severity. Furthermore, we demonstrated that being in the highest SII quartile doubles the risk of developing severe DKA. We determined a cut-off value of 2524.24 for SII to predict DKA severity with high specificity.

DKA, the most common hyperglycemic emergency, is caused by insulin deficiency and is associated with a rise of counterregulatory hormones such as glucagon, cortisol, and catecholamines. This hormonal disturbance and the accompanying inflammatory response are the fundamental mechanisms implicated in DKA pathogenesis. DKA is associated with substantial morbidity, especially in its severe form^1^. Early recognition of severe DKA and the timely initiation of therapy will correct acidemia, bring back the circulatory volume, and normalize levels of inflammatory and oxidative stress markers. This is vital to improve the outcomes^21^. There is a concord between different guidelines that the severity of DKA is determined mainly by the degree of acidemia (PH and HCO_3_), not by the degree of ketonemia or hyperglycemia^1^. Despite of being the gold standard test to define acid-base status, the analysis of blood gases is not widely available and has many limitations of the procedure or the interpretation of its results^22^. Preferably, we need a predictor from the patient’s routine lab that can predict the severity of DKA. CBC is a simple, convenient, and economical test that was formerly investigated for its ability to predict DKA severity.

Leukocytosis was more prevalent among patients with severe DKA in our study. WBC count is well-known to increase with DKA due to the associated pro-inflammatory state, elevated cortisol levels, and stimulation of the sympathetic nervous system^5^. However, leukocytosis was reported in DKA with and without infection, which makes it a less sensitive marker of severity. Besides, leukocytosis is a non-specific acute phase response and can’t account for the changes in white cell subtypes, so the value of using leukocytosis in predicting DKA severity is limited^7,23^.

We did not find a higher prevalence of elevated NLR or PLR in the severe DKA group, nor a correlation with DKA markers of severity that is PH and HCO_3_. Also, higher NLR or PLR were not risk factors for severe DKA. Combined markers such as NLR and PLR have been explored in DKA states. It was formerly reported that among adult T1DM patients, NLR positively correlated with creatinine and HbA1c levels, and negatively correlated with albumin level after adjusting for age and duration of diabetes. Also, NLR is associated with the occurrence of DKA in T1DM patients without infections^8^. Among children with severe DKA, NLR is associated with a higher risk of cerebral edema (*P*=0.045)^24^. A study on PLR proved an association with increased 90-day readmission and mortality rates amongst DKA patients admitted to ICU. However, the study did not include patients who were managed in hospital wards or triage areas^25^. To the best of our knowledge, no study has reported the capability of PLR or NLR to differentiate severe and non-severe DKA among adult T1DM patients.

As a combined ratio based on platelets, neutrophils, and lymphocytes, the SII correlated with severity in our study. This can be explained by the role of inflammation and immune regulation in the pathogenesis of DKA. The hyperglycemic crisis of DKA markedly elevates plasma levels of cytokines, adhesion molecules, and reactive oxygen species (ROS). The net result would be a pro-inflammatory-proadhesive phenotype leading to cellular activation, adhesion, endothelial and oxidative damage^5,6^. DKA results in increased neutrophil counts and neutrophil-mediated inflammatory state^7^. Lymphocytes have regulatory and protective effects. DKA-induced increase in ROS can damage the lymphocytes and lead to their apoptosis. The balance between neutrophils and lymphocytes reflects the interaction between the inflammatory activator and controller elements. Among DKA patients, a more severe inflammatory response is characterized by a higher neutrophil and a lower lymphocyte count^8^. Also, DKA status is associated with increased platelet counts compared to non-DKA status^26^. The proposed underlying mechanism is also related to the pro-inflammatory milieu of DKA^25^. Platelets contain pro-inflammatory molecules that modulate the immune and inflammatory responses. In diabetic patients, there is dysregulation of some signaling pathways of platelets^27^. Accordingly, a more severe DKA is expected to be associated with high SII reflecting a varying degree of the combination of thrombocytosis, neutrophilia, and/or lymphopenia. This finding was confirmed in our study, where higher SII was independently linked to severe DKA and correlated with other markers of severity that is PH and HCO_3_.

SII is an integrated ratio that was originally used to prognosticate patients with hepatocellular carcinoma^12^. It is considered to reflect immune-inflammation status better than NLR and PLR^28^. A study reported increased SII among patients with new-onset diabetes secondary to pancreatic ductal adenocarcinoma suggesting an inflammatory component as an underlying pathophysiological mechanism of hyperglycemia. The authors reported also a significant correlation between SII and insulin resistance^29^. Analysis of data from the National Health and Nutrition Examination Survey (NHANES) database demonstrates that higher SII is associated with increased prevalence of diabetes, and that each additional unit of SII increases the possibility of having diabetes by 4%^30^. Among diabetic patients, higher SII is associated with an increased likelihood of diabetic kidney disease, depression, and recurrent atrial fibrillation after ablation^31–33^.

We found that being in the fourth SII quartile (SII ≥ 2105) was an independent risk factor for severe DKA. We also found that an SII cut-off value of 2524.24 may predict severe DKA with high specificity. The optimal cut-off value for SII associated with different diseases is debatable. A study found that SII greater than or equal to 445.21 was associated with diabetic kidney disease^31^. Another study found that SII of 444.77 can predict atrial fibrillation recurrence after ablation among diabetic patients^33^. In studies that included diabetic and non-diabetic patients, SII greater than 2140 could predict poor outcomes in stroke patients after endovascular thrombectomy^34^. Also, SII greater than 2120 was an independent risk factor of mortality among patients with ischemic stroke in another study^35^. Different cut-off values that can predict outcomes among patients with ASCVD were described in two meta-analyses^14,17^.

During the past years, combined CBC immune-inflammation markers such as PLR and PLR have been studied considerably in different clinical settings. SII is an innovative biomarker of the systemic immune-inflammatory response. It links three blood cell elements to a metabolic hyperglycemic emergency highlighting the role of inflammation in DKA. As an integrated biomarker, SII can provide more precise information than one or two types of blood cells.

To the best of our knowledge, this is the first study demonstrating the association between SII and DKA severity in T1DM. Although our study presented novel findings, it has some limitations. First, it represents preliminary evidence, and further research is needed to confirm these findings. Second, the observational nature of the study has its limitations although we tried to reduce bias by applying rigorous inclusion and exclusion criteria. Finally, we measured SII only on admission, and no serial measurements were done.

## Conclusion

In conclusion, SII either as a categorical or continuous variable correlated with an increased DKA severity. It can be readily obtained from CBC and could be a valuable and straightforward approach to assessing the severity of DKA among T1DM patients without infection. Although SII cannot replace conventional PH criteria, it can add complementary data to the prediction models of DKA severity. The identification of the link between SII and DKA severity can be important for the early recognition, timely treatment, and proper recovery of patients. Further research is needed to uncover the underlying pathogenic mechanisms and the potential impact on patients’ outcomes.

## Ethical approval

The study was approved by Jahra Hospital research committee with approval number (J1 –18072023).

## Consent

Waived by the IRB (Jahra Hospital research committee, J1–18072023) due to the retrospective nature of the study and the data anonymity.

## Sources of funding

This research received no external funding.

## Author contribution

M.A.: conceptualization, data curation, methodology, project administration, supervision, writing—original draft. A.H.A.: data curation, formal analysis, supervision, writing—review and editing. A.A.: data curation, methodology, project administration, supervision, writing—review and editing. A.A.: data curation, investigation. F.A.: data curation, investigation. M.A.: data curation, investigation. A.A.: data curation, investigation. B.B.N.: data curation, investigation. A.A.: data curation, investigation. O.A.A.: data curation, formal analysis, writing—review and editing.

## Conflicts of interest disclosure

The authors declare no conflicts of interest.

## Research registration unique identifying number (UIN)

1. The study is registered at https://www.clinicaltrials.gov/ with registration ID (NCT06251895).
2. https://register.clinicaltrials.gov/prs/app/action/SelectProtocol?sid=S000E3NU&selectaction=Edit&uid=U0007CEZ&ts=2&cx=-afwzug.

## Guarantor

Mohamed Aon.

## Data availability statement

Datasets analyzed during the current study are available upon reasonable request from the corresponding author and after IRB approval.

## Provenance and peer review

Not commissioned, externally peer-reviewed.

## Supplementary Material

SUPPLEMENTARY MATERIAL
