# Supplementary material for: Association of the systemic immune-inflammation index (SII) and severity of diabetic ketoacidosis in patients with type 1 diabetes mellitus: a retrospective cohort study
Source: Ann Med Surg (Lond). 2024 May 20;86(7):3865–72. doi: 10.1097/MS9.0000000000002185 (PMC11230746; doi:10.1097/MS9.0000000000002185)
Supplement: SUPPLEMENTARY MATERIAL [file ms9-86-3865-s002.pdf]

Supplementary table 1: Univariate and multivariate logistic regression for the potential risk factors of DKA severity.						
	Model 1		Model 2		Model 3	
Variables	Odds ratio (95% CI)	P-value	Odds ratio (95% CI)	P-value	Odds ratio (95% CI)	P-value
Age	0.988 (0.962 – 1.02)	0.396	-		-	
Sex						
Male	Reference	Reference	-		-	
Female	0.999 (0.626 – 1.59)	0.996	-		-	
Comorbidities						
Hypothyroidism	0.328 (0.045 – 1.66)	0.202	-		-	
Others	0.587 (0.110 – 2.61)	0.481	-		-	
NLR quartiles						
Q1	Reference	Reference	-		-	
Q2	0.935 (0.481 – 1.82)	0.844	-		-	
Q3	1.010 (0.520 – 1.96)	0.977	-		-	
Q4	1.725 (0.890 – 3.37)	0.108	-		-	
PLR quartiles						
Q1	Reference	Reference	-		-	
Q2	1.18 (0.611 – 230)	0.618	-		-	
Q3	1.07 (0.536 – 2.14)	0.846	-		-	

Q4	1.49 (0.754 – 2.94)	0.253	-		-	
Platelets (*10^9/L)	1.00 (1.00 – 1.01)	**<0.001**	1.00 (1.002 – 1.01)	**<0.001**	1.005 (1.002 – 1.01)	**0.002**
WBCs (*10^9/L)	1.13 (1.07 – 1.19)	**<0.001**	1.127 (1.069 – 1.19)	**<0.001**	1.140 (1.070 – 1.22)	**<0.001**
Neutrophils (*10^9/L)	1.08 (1.03 – 1.13)	**0.004**	1.074 (1.025 – 1.13)	**0.005**	1.075 (1.022 – 1.14)	**0.014**
Lymphocytes (*10^9/L)	1.09 (0.963 – 1.28)	0.196	**-**		**-**	
SBP	0.999 (0.982 – 1.02)	0.949	**-**		**-**	
DBP	0.955 (0.925 – 0.985)	**0.004**	0.957 (0.926 – 0.987)	**0.006**	0.955 (0.92 – 0.989)	**0.012**
Pulse (bpm)	1.03 (1.01 – 1.04)	**<0.001**	1.026 (1.010 – 1.04)	**0.002**	1.022 (1.005 – 1.04)	**0.014**
Temperature (°C)	0.676 (0.424 – 1.07)	0.097	**-**		**-**	
HbA1c (%)	1.10 (0.991 – 1.23)	0.075	**-**		**-**	
Glucose (mmol/L)	1.03 (1.00 – 1.06)	**0.033**	1.029 (1.002 – 1.06)	**0.038**	1.015 (0.981 – 1.05)	0.399*
Hemoglobin (g/L)	1.00 (0.999 – 1.01)	0.100	**-**		**-**	
Urea (mmol/L)	1.11 (1.01 – 1.23)	**0.036**	1.155 (1.032 – 1.30)	**0.013**	1.107 (0.918 – 1.34)	0.288*
Creatinine (umol/L)	1.01 (0.955 – 1.02)	0.255	**-**		**-**	
AST (IU/L)	1.00 (1.00 – 1.01)	0.105	**-**		**-**	
ALT (IU/L)	1.00 (0.999 – 1.01)	0.200	**-**		**-**	
PCT (ng/ml)	1.08 (0.791 – 1.48)	0.637	**-**		**-**	

NLR quartiles: Q1 <1.87 (n= 60); Q2 = 1.87 – <3.26 (n= 60); Q3 = 3.26 – <6.06 (n= 60); Q4 ≥ 6.06 (n= 61).
PLR quartiles: Q1 <118 (n= 58); Q2 = 118 – <159 (n= 62); Q3 = 159 – <230 (n= 59); Q4 ≥ 230 (n= 62).
Model 1: Unadjusted odds ratio (OR).
Model 2: Adjusted to age, sex, and comorbidities.
Model 3: Adjusted to age, sex, comorbidities, blood glucose, HbA1c, creatinine, urea, AST, ALT, and PCT.
*Model 3 except for the investigated variable.
Abbreviations: ALT, alanine transaminase; AST, aspartate transaminase; bpm, beat per minute; DBP, diastolic blood pressure; DKA, Diabetic ketoacidosis; HbA1c, glycated hemoglobin; NLR, neutrophil lymphocyte ratio; PCT, procalcitonin; PLR, platelet lymphocyte ratio; SBP, systolic blood pressure; SII, systemic immune-inflammation index; WBCs, white blood cells.
