# Supplementary material for: Association of the systemic immune-inflammation index (SII) and severity of diabetic ketoacidosis in patients with type 1 diabetes mellitus: a retrospective cohort study
Source: Ann Med Surg (Lond). 2024 May 20;86(7):3865–72. doi: 10.1097/MS9.0000000000002185 (PMC11230746; doi:10.1097/MS9.0000000000002185)
Supplement: SUPPLEMENTARY MATERIAL [file ms9-86-3865-s003.pdf]

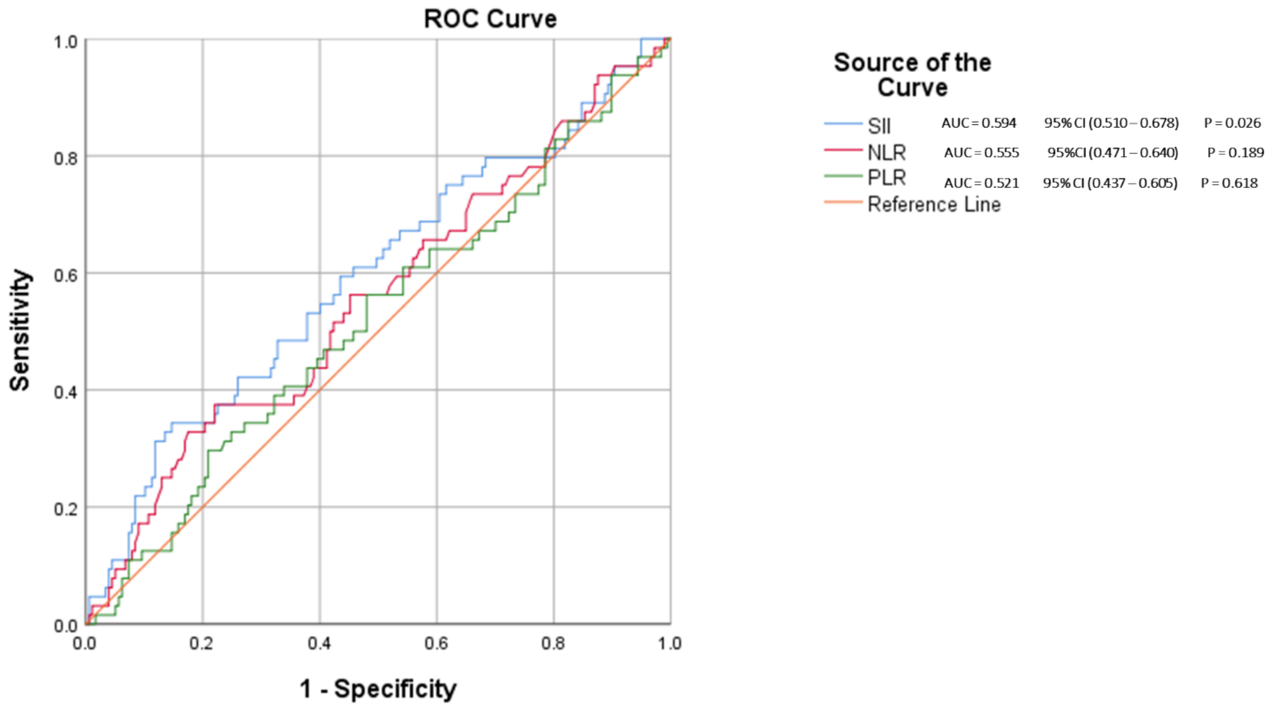

Supplementary Figure 1: ROC curve for SII, NLR, and PLR.

Abbreviations: NLR, neutrophil lymphocyte ratio; PLR, platelet lymphocyte ratio; ROC, receiver operating characteristics; SII, systemic immune-inflammation index.
